# Estrogens and Glucocorticoids in Mammary Adipose Tissue: Relationships with Body Mass Index and Breast Cancer Features

**DOI:** 10.1210/clinem/dgz268

**Published:** 2019-12-20

**Authors:** Sofia Laforest, Mélissa Pelletier, Nina Denver, Brigitte Poirier, Sébastien Nguyen, Brian R Walker, Francine Durocher, Natalie Z M Homer, Caroline Diorio, Ruth Andrew, André Tchernof

**Affiliations:** 1 CHU de Québec-Université Laval Research Center (Endocrinology and Nephrology division), School of Nutrition, Faculty of Agriculture and Food Sciences, Université Laval, Québec, Canada; 2 Institut universitaire de cardiologie et de pneumologie de Québec, Université Laval, Québec, Canada; 3 Mass Spectrometry Core, Edinburgh Clinical Research Facility, Queen’s Medical Research Institute, Edinburgh, UK; 4 Institute of Cardiovascular and Medical Sciences, College of Medical, Veterinary and Life Sciences, University of Glasgow, University Avenue, Glasgow, UK; 5 CHU de Québec-Université Laval Research Center (Oncology division), Université Laval Cancer Research Center and Department of Surgery, Faculty of Medicine, Université Laval, Québec, Canada; 6 Centre des maladies du sein Deschênes-Fabia, Hôpital Saint-Sacrement, Québec, Canada; 7 University/BHF Centre for Cardiovascular Science, Queen’s Medical Research Institute, University of Edinburgh, Edinburgh, UK; 8 Institute of Genetic Medicine, Newcastle University, Newcastle upon Tyne, UK; 9 CHU de Québec-Université Laval Research Center (Endocrinology and Nephrology division), Université Laval Cancer Research Center and Department of Molecular Medicine, Faculty of Medicine, Université Laval, Québec, Canada; 10 CHU de Québec-Université Laval Research Center (Oncology division), Université Laval Cancer Research Center and Department of Social and Preventive Medicine, Faculty of Medicine, Université Laval, Québec, Canada

**Keywords:** estradiol, estrone, cortisol, cortisone, breast cancer, adiposity

## Abstract

**Context:**

Adipose tissue is an important site for extragonadal steroid hormone biosynthesis through the expression and activity of P450 aromatase, 11β-hydroxysteroid dehydrogenase (HSD) 1, and 17β-HSDs. The contribution of steroid hormones produced by adjacent adipose tissue for the progression and survival of breast tumors is unknown.

**Objective:**

To quantify estrogens (estradiol, estrone) and glucocorticoids (cortisol, cortisone) in breast adipose tissue from both healthy and diseased women and their relationships with adiposity indices and breast cancer prognostic markers.

**Design and setting:**

Breast adipose tissue was collected at time of surgery.

**Patients:**

Pre- and postmenopausal women undergoing partial mastectomy for treatment of breast cancer (n = 17) or reduction mammoplasty (n = 6) were studied.

**Interventions:**

Relative estrogen and glucocorticoid amounts were determined by liquid chromatography tandem mass spectrometry.

**Results:**

The targeted steroids were reliably detected and quantified in mammary adipose tissues. Women with ER+/PR+ tumor had higher relative estradiol amount than women with ER–/PR– tumor (*P* < .05). The ratio of estradiol-to-estrone was higher in lean women than in women with a body mass index (BMI) ≥ 25 kg/m^2^ (*P* < .05). Mixed-model analyses showed that estradiol, cortisone, and cortisol were negatively associated with tumor size (*P* < .05). Relationships between glucocorticoids and tumor size remained significant after adjustment for BMI. The cortisol-to-cortisone ratio was negatively associated with tumor stage (*P* < .05) independently of BMI.

**Conclusions:**

We reliably quantified estrogens and glucocorticoids in breast adipose tissue from healthy women and women suffering from breast cancer. Our findings suggest that smaller breast tumors are associated with higher relative amounts of estradiol and cortisol in adipose tissue.

Being overweight or obese is a well-known risk factor for postmenopausal breast cancer (1, 2). Obesity is also linked to a poorer prognosis in women with breast cancer regardless of their menopausal status (1, 3, 4). Women with obesity have more aggressive tumors, higher mortality rates and incidence of metastases, and increased risk of recurrence (1, 5–8). Central obesity, as measured by waist circumference (WC), is an emerging risk factor for both pre- and postmenopausal breast cancer (9). Furthermore, the efficacy of chemotherapy, radiotherapy, surgery, and endocrine therapy is reduced in women with obesity and possibly more so with increased visceral fat accumulation (10–14). The mechanisms underlying higher risk and reduced treatment efficacy are not fully understood. Altered secretion of adipokines, growth factors, and steroids by dysfunctional mammary adipose tissue may contribute to a proinflammatory, growth-promoting microenvironment for cancer cells (15). Recent evidence from human studies has shown that local breast adipose tissue does present an altered biological profile, as described above, concomitant with body mass index (BMI) increases (16–21). Reports from Dannenberg and collaborators have shown that mean adipocyte cell size from breast adipose tissue was positively associated with BMI and increased aromatase expression and inflammation markers such as crown-like structures and was also related to menopausal status (19, 20).

Expression and activity of several steroidogenic enzymes present in adipose tissue have been linked to increased adiposity (22). For example, higher rates of androgen-to-estrogen conversion through aromatization in adipose tissue (22) have been proposed as a mechanism for the obesity-related increase in breast cancer risk. Findings from our group suggest that known estrogenic 17β-hydroxysteroid dehydrogenase (HSD) (type 1, 7, and 12)-mediated conversion of estrone (E1) to estradiol (E2) is 5 times higher in differentiated adipocytes than in preadipocytes (23). Increased mean adipocyte size is associated with higher expression and activity of 11β-HSD type 1, which locally converts cortisone to active cortisol through oxoreductase activity (24, 25).

Despite their well-known anti-inflammatory effects, glucocorticoids (GCs) could contribute to breast cancer initiation, progression, and survival via the activation of the glucocorticoid receptor (GR) or by increasing aromatase expression via the GC response element (GRE) on exon I.4 of the *CYP19A1* gene (26). Moreover, *HSD11B1* expression increases with estrogen receptor β (ERβ) activation (27). In a rodent model of breast cancer, increased GC nurtured the transition from DCIS (ductal carcinoma in situ) to IDC (invasive ductal carcinoma) while administration of RU-486 was able to partially block this effect, that is, prevent breast cancer progression to IDC (28). Considering the slow turnover of GCs in adipose tissue (29), there is biological plausibility for autocrine and paracrine effects of active GCs, such as cortisol, in the tumoral microenvironment.

Hence, steroid dynamics in breast adipose tissue and cancer appear to involve more than overexpression and increased activity of aromatase. We currently have very little information about the relative importance of adipose tissue dysfunction markers such as altered steroid conversion to tumor progression and aggressiveness or patient prognosis. Moreover, considering the complexity of potential enzymatic hormone conversion in adipose tissues, direct measurement of active hormones and their precursors has become highly relevant.

Liquid chromatography followed by tandem mass spectrometry (LC-MS/MS) is recognized as the gold standard to quantify endogenous steroids in plasma; however, tissue steroids are more difficult to measure than those in plasma due to the complexity of the matrix and the need to homogenize uniformly. Measurements are confounded by the low abundance and the poor ionization profile of steroids as well as the higher concentrations of lipids in adipose tissue than in plasma, which result in higher susceptibility of so-called matrix effects. We aimed to characterize the relationship of local cortisol and E2 as well as inactive steroid cortisone and main E2 precursor E1 with adiposity and prognostic markers in a sample of women with or without breast cancer. We hypothesize that independently of menopausal status (1) relative E2 and cortisol breast adipose tissue amounts as well as the ratio E2:E1 and cortisol on cortisone are increased with adiposity; (2) worst clinical breast cancer features, such as tumor stage, size and grade are associated with lower relative adipose tissue steroid amounts; and (3) relative estrogen amounts are increased in women with ER+/PR+ tumors. We also investigated if these relationships were independent of total adiposity, that is, reflecting the microenvironment or a characteristic of the adiposity state.

## Methods

### Study sample and data collection

The study protocol was approved by the Research Ethics Committees of Laval University Medical Center (DR-002-136). All patients signed a written, informed consent prior to surgery. Breast adipose tissue was obtained from women undergoing partial or total mastectomy for treatment of breast cancer (n = 17) or reduction mammoplasty (n = 6). Fresh tissue specimens were acquired from residual resected breast tissues that were not required for clinical diagnosis at least 1 cm away from the tumor margins. Information on clinicopathologic and anthropometric factors was collected from in-person interviews, phone call interviews, and/or medical records.

### Laboratory methods

#### Cell sizing.

Breast adipocyte size was measured as previously described (30, 31) in formalin-fixed adipose tissue. Briefly, 250 breast adipocytes from 10 randomly chosen areas at ×40 magnification using Calopix software (Tribvn) were measured for each tissue sample in a blinded fashion.

### Liquid chromatography-tandem mass spectrometry

#### Standards and solvents.

E1 and E2 were obtained from Steraloids, Inc. (Newport, RI). Cortisone, cortisol, and iodomethane (≥99%) were from Sigma-Aldrich, Inc. (Dorset, UK). 1-(2,4-dinitro-5-fluorophenyl)-4-methylpiperazine (PPZ) was obtained from TCI chemicals (Chuo-ku, Tokyo, Japan). High-performance liquid chromatography (HPLC) grade glass distilled solvents (acetone; ethyl acetate [EtOAc]; water) were from Fisher Scientific UK Limited (Loughborough, UK). Analytical reagent (AR) grade ethanol (EtOH) and HPLC grade glass distilled solvents (acetonitrile; methanol [MeOH]), and LCMS grade (acetonitrile [(CH_3_)_2_CO]; formic acid [FA; ≥98%]; water) solvents were from VWR (Lutterworth, UK).

#### Instrumentation.

Cortisone, cortisol, E1, and E2 were measured by LC-MS/MS, using a UHPLC Shimadzu Nexera X2 system (UK) coupled to a Sciex QTRAP^®^ 6500+ (SCIEX, Warrington, UK) equipped with an electrospray ionization interface (ESI). Mass spectrometry conditions were previously described in conjunction with ion spray voltage (5500 V) and source temperature (500°C) (32).

#### Sample preparation.

Following enrichment of frozen adipose tissue samples (~200 mg) with 3 internal standards (IS), 9,11,12,12-[^2^H_4_]-cortisol (D_4_F5 ng; Cambridge Isotopes Laboratory, Thaxted, UK), 2,3,4-[^13^C_3_]-17β-estradiol and 2,3,4-[^13^C_3_]-estrone (^13^C_3_E2, ^13^C_3_E1 respectively; 5 ng; Sigma-Aldrich, Inc., St. Louis, MO), analytes were extracted as described below.

Briefly, frozen adipose tissue samples were homogenized (Model Pro 200, ProScientific, Inc, Monroe, CT) in EtOH:EtOAc (1 mL; 1:1) and immediately frozen on dry ice and stored at –80°C overnight. The following morning, samples were thawed on wet ice and sonicated (8 × 15-second bursts with 1-minute gaps; Ultrasonic cleaner, Branson Ultrasonic Inc, Danbury, CT). Samples were subjected to centrifugation (3200*g*, 45 minutes, 4°C; Heraeus Megafuge 16R, ThermoFisher Scientific, Germany). The supernatant was transferred into a new glass tube and dried down under oxygen-free nitrogen (OFN; 60°C). Samples were resuspended in aqueous MeOH (30% v/v, 5 mL). Solid-phase extraction was carried out after conditioning C18 Sep-Pak columns (12 cc, 2 g; Waters, Wilmslow, UK; MeOH (2 × 10 mL), followed by H_2_O (2 × 10 mL)). The adipose extract was loaded, and the column was washed with H_2_O (10 mL) followed by aqueous MeOH (5%, 10 mL). Steroids were eluted with MeOH:CH_3_CN (1:1, 10 mL). The eluent was dried down under OFN at 60°C prior to derivatization of estrogens. Generation of 1-(2,4-dinitrophenyl)-4,4-dimethylpiperazinium (MPPZ) derivatives of E1 and E2 has already been described (32–34). Derivatization was carried out by incubating (1 hour, 60°C) with [(CH_3_)_2_CO] (70 µL), NaHCO_3_ (10 µL, 1M, Sigma-Aldrich, Inc., St. Louis, MO) and PPZ (10 µL, 1 mg/mL, dissolved in (CH_3_)_2_CO) followed by the addition of CH_3_I (100 µL), and further incubation (2 hours, 40°C) as previously described (33, 34). Samples were dissolved in H_2_O:CH_3_CN (70 µL; 70:30) and transferred to LC vials.

#### Liquid chromatography parameters.

Following injection (30 µL), analytes were separated on an ACE 2 Excel C18-PFP (150 × 2.1 mm, 2 μm; HiChrom, Reading, UK, 40°C) column. The elution process started with mobile phase compositions of 90:10 H_2_O with 0.1% FA (solution A) and CH_3_CN with 0.1% FA (solution B) which was maintained for 1 minute. This was followed by an 11-minute linear gradient to 50% solution B, which was maintained for 2 minutes, before returning to 10% solution B at 15 minutes and maintained for 3 minutes, all at a constant flow rate of 0.5 mL/min.

#### Linearity and lower limit of quantitation.

Blank samples and aliquots containing estrogens (5, 7.5, 10, 15, 25, 50, 100, 200, 500, 1000 pg/sample), GCs (50, 75, 100, 150, 250, 500, 1000, 2000, 5000, 10 000 pg/sample), and IS (500 pg) were analyzed by LC-MS/MS. Calibration curves were plotted as the peak area ratio (standard/IS) versus amount of analytes (GCs or estrogens). Calibration lines of best fit were acceptable if the regression coefficient, r, was >0.98. Weightings of 1/x were used for all 4 steroids. Linearity and lower limit of quantitation LLOQs were 50 pg, 15 pg, 100 pg, and 75 pg for E2, E1, cortisol, and cortisone, respectively. Values below the confirmed LLOQ were calculated as LLOQ divided by 3: the lowest acceptable signal-to-noise ratio. The values were then converted to pmol/kg according to the weight of the corresponding adipose tissue sample. This transformation was performed to avoid null values to calculate steroid ratios as described below.

### Calculated ratios

All steroid amounts were converted into pmol/kg. Those values were then used to calculate product and substrate ratios, as described below. The ratio of cortisol:cortisone was used as a marker of 11β-HSD1 enzyme activity. The ratio of E2:E1 was used as a marker of estrogenic 17β-HSD enzyme activity.

### Gene expression

Tissues were homogenized in Qiazol buffer (Qiagen, Germantown, MD, USA) and total RNA was extracted using the RNeasy mini kit on-column DNase (Qiagen, Hilden, DE) treatment following the manufacturer’s instructions. First-strand cDNA synthesis was accomplished using 1 µg of RNA in a reaction containing 200 U of Superscript IV Rnase H-RT (Invitrogen Life Technologies, Burlington, Canada). cDNA corresponding to 20 ng of total RNA was used to perform fluorescent-based real-time polymerase chain reaction (PCR) quantification using the LightCycler 480 (Roche Diagnostics, Mannheim, DE). Reagent LightCycler 480 SYBRGreen I Master (Roche Diagnostics, Indianapolis, IN) was used as described by the manufacturer. The conditions for PCR reactions were 45 cycles, denaturation at 95°C for 10 seconds, annealing at 58–60°C for 10 seconds, elongation at 72°C for 14 seconds, and then 72°C for 5 seconds (reading). Oligoprimer pairs were designed by GeneTool 2.0 software (Biotools Inc., Edmonton, Canada) and their specificity was verified by blast in the GenBank database. The synthesis was performed by IDT (Integrated DNA Technology, Coralville, IA) (Table 1). Normalization was performed using the following reference genes: ATP synthase O subunit (*ATP5O*), hypoxanthine guanine phosphoribosyl transferase 1 (*HPRT1*), and glyceraldehyde-3-phosphate dehydrogenase (*GAPDH*). Quantitative real-time PCR measurements were performed by the CHU de Québec Research Center (CHU) Gene Expression Platform, Quebec, Canada, and were compliant with the Minimum Information for Publication of Quantitative Real-Time PCR Experiments (MIQE) guidelines.

**Table 1.** Sequence primers and gene description.

Gene symbol	Description	GenBank	size (pb)	Primer sequence 5′→3′ S/AS
*HSD11B1*	*Homo sapiens* hydroxysteroid 11-β dehydrogenase 1 (*HSD11B1*), 3 transcripts	NM_005525	85	TGTGCCCTGGAGATCATCAAA/ TGATCAGAAGAGTGGTCCAGAGTG
*CYP19A1*	*Homo sapiens* cytochrome P450, family 19, subfamily A, polypeptide 1 (*CYP19A1*), 11 transcripts	NM_000103	123	AAGAGGCAATAATAAAGGAAATCCAGAC/ CGACAGGCTGGTACCGCATGCTC
*HSD17B12*	*Homo sapiens* hydroxysteroid (17- β) dehydrogenase 12 (*HSD17B12*)	NM_016142	145	CCCACTCTTGACCATCTATTCTG/ CTTCCGGATTTTAGCCAGTTTTGTA
*HSD17B7*	*Homo sapiens* hydroxysteroid (17- β) dehydrogenase 7 (*HSD17B7*)	NM_016371	293	TCCACCAAAAGCCTGAATCTCTC/ GGGCTCACTATGTTTCTCAGGC
*ESR1*	*Homo sapiens* estrogen receptor 1 (*ESR1*), 6 transcripts	NM_000125	293	TGCAAAATCTAACCCCTAAGGAAGTG/ CTCCCAGTACCCACAGTCCATCTC
*ESR2*	*Homo sapiens* estrogen receptor 2 (*ESR2*), 5 transcripts	NM_001437	114	ACGCCGTGACCGATGCTTTGG/ TCGCATGCCTGACGTGGGACA
*ATP5O*	*Homo sapiens* ATP synthase, H^+^ transporting, mitochondrial F1 complex, O subunit (*ATP5O*)	NM_001697	267	ATTGAAGGTCGCTATGCCACAG/ AACGACTCCTTGGGTATTGCTTAA
*HPRT1*	*Homo sapiens* hypoxanthine phosphoribosyltransferase 1 (*HPRT1*)	NM_000194	157	AGTTCTGTGGCCATCTGCTTAGTAG/ AAACAACAATCCGCCCAAAGG
*GAPDH*	*Homo sapiens* glyceraldehyde-3-phosphate dehydrogenase (*GAPDH*)	NM_002046	194	GGCTCTCCAGAACATCATCCCT/ ACGCCTGCTTCACCACCTTCTT

### Statistical analyses

Differences in breast adipocyte diameter between women with breast cancer (cases) and women without breast cancer (controls) or according to menopausal status were assessed by Student’s t-test. Cell size frequency distribution differences between case and control women or according to menopausal status were assessed by the Kolmogorov–Smirnov (KS) test. Women were subdivided in categories of BMI (lean [<25 kg/m^2^] or overweight and obese [≥25 kg/m^2^]) or according to their estrogen and progesterone receptor (ER/PR) status. Relative hormone amounts or ratios between those subgroups were assessed by Student’s t-test. Satterthwaite approximation was used when variances were deemed unequal according to a conservative folded F statistic (*P* < .10). Exact *P*-values computed using nonparametric Wilcoxon tests showed similar results. Women were subdivided according to their respective tumor size, according to tertiles of the distribution. A mixed model was performed to evaluate the relationship between relative hormone amounts and tumor size (in tertiles or continuous; as determined by the best AIC fit for the model), grade (categorical), and stage (categorical). A repeated statement was incorporated into the model to account for the nonconstant variance among the residuals, that is, specifying a variance component covariance structure in the model. Non-normally distributed variables were log-transformed to achieve normality and linearity. Models were further adjusted for BMI (as a continuous variable) to account for total adiposity. Adjustments for menopausal status and current use of hormonal derivatives (as combined indicator variables) were also performed as they are identified as confounders in the breast cancer literature. Spearman correlation coefficients were computed to assess the relationship between relative steroid amount, prognostic factors, and relative expression of genes. *P* < .05 was considered significant. All statistical analyses were performed with SAS software (SAS Institute, Cary, NC).

## Results

Table 2 shows the characteristics of the study sample. Women were overweight with a median BMI of 25.6 kg/m^2^ and a median age of 55 years. Postmenopausal status was equally balanced across case and control women. Table 3 presents the clinicopathological features of premenopausal and postmenopausal breast cancer patients. Most women presented with a unilateral breast lesion. Breast tumors were mainly of ductal histology (82%). Only 1 woman presented a HER2+ tumor and 13 women had an ER+/PR+ tumor. Half of the women had stage 2 breast cancer as classified by TNM score (35).

**Table 2.** Clinical characteristics of the women with breast cancer and control women.

	Women		
Variables (median, [Q1-Q3])	All (n = 23)	Controls (n = 6)	Cases (n = 17)
Age (years)	55.0 (50.1–62.9)	53.7 (44.9–57.5)	55.9 (53.2–63.2)
Menopausal status			
Premenopausal n (%)	8 (35)	2 (33)	6 (35)
Postmenopausal n (%)	15 (65)	4 (67)	11 (65)
Anthropometrics			
BMI (kg/m^2^)	25.6 (24.3–28.2)	27.1 (24.3–29.4)	25.4 (24.5–26.8)
WC (cm)	−	−	94 (86–99)
Breast adipocyte mean diameter (µm)	75.5 (67.3–87.5)^*a*^	87.9 (86.2–89.5)	74.1 (66.1–80.3)^*b*^
Hormonal derivatives			
Current oral contraceptive use (yes) n (% of premenopausal)	6 (75)	1 (50)	5 (83)
Current HRT use (yes) n (% of postmenopausal)	5 (33)	2 (50)	3 (27)

Abbreviations: BMI, body mass index; HRT, hormonal replacement therapy; WC, waist circumference.

^*a*^n = 22, ^*b*^n = 16.

**Table 3.** Characteristics of the tumor.

Characteristics n (%)	Premenopausal (n = 6)	Postmenopausal (n = 11)	All (n = 17)
Lesion			
Unilateral	5 (83.33)	10 (90.91)	15 (88.24)
Bilateral	1 (16.67)	1 (9.09)	2 (11.76)
Histology			
Ductal	6 (100.00)	8 (72.73)	14 (82.35)
Others^*a*^	0 (0.00)	3 (27.27)	3 (17.65)
Receptor status			
ER+/PR+	5 (83.33)	8 (72.73)	13 (76.47)
TNM status			
0	0 (0.00)	3 (27.27)	3 (17.65)
1	2 (33.33)	4 (36.36)	6 (35.29)
2	4 (66.67)	4 (36.36)	8 (47.06)

Abbreviations: ER, estrogen receptor; PR, progesterone receptor.

^*a*^Includes lobular, mucinous, and metaplastic carcinomas.

**Figure 1.**
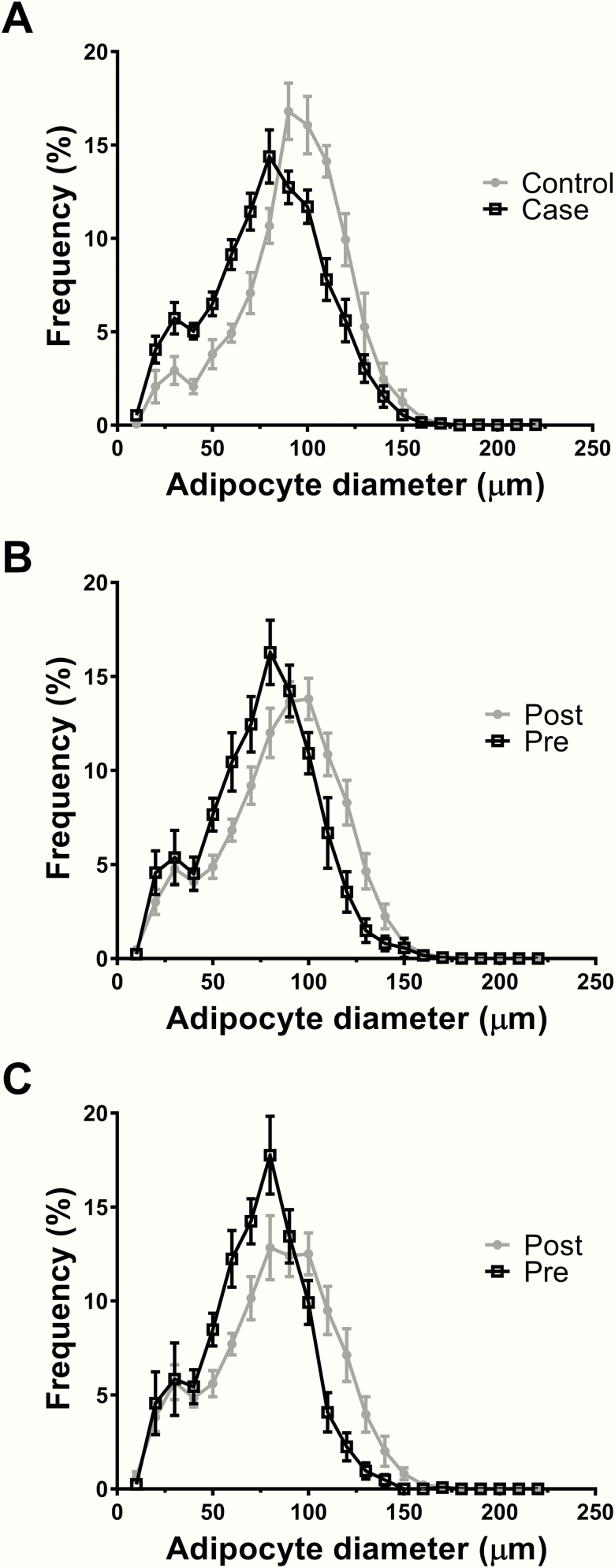
Size distribution of breast adipocytes. (A) Comparison between control and case women (t-test, *P* = .0072; KS, *P* = .0041; n = 22). (B) Comparison between premenopausal and postmenopausal women (t-test, *P* = .0510; *KS*, *P* = .0864; n = 22). (C) Comparison between premenopausal and postmenopausal women with cancer (t-test, *P* = .0552; *KS*, *P* = .0480; n = 16).

Women with or without breast cancer were comparable for primary clinical characteristics, except for mean fat cell size which was 13.3 µm higher in the control women (t-test, *P* = .0072). This difference was also shown by the adipocyte size distribution curve comparison (KS, *P* = .0041) (Fig. 1A). Postmenopausal women had a 9.7 µm higher mean adipocyte size than premenopausal women as well as a right-shifted adipocyte size distribution; however, this difference was not statistically significant (Fig. 1B). The distribution remained similar when considering only cancer cases (Fig. 1C).

### Quantification of steroids

Table 4 shows relative adipose tissue steroid amounts and calculated ratios. Relative E1 and cortisol amounts were quantified in each of the 23 samples of mammary adipose tissue. E2 was below the limit of quantification for 2 samples. Cortisone was below the limit of quantification for 5 samples, 4 of which were obtained from control women.

**Table 4.** Relative amounts of steroid and associated ratios in adipose tissue.

	Women			
Steroids (median, [Q1-Q3])	All (n = 23)	Controls (n = 6)	Cases (n = 17)	*P-value
Cortisone (pmol/kg)	5501 (1829–16 748)	326 (298–1829)	7817 (2811–16 748)	.0055
Cortisol (pmol/kg)	22 671 (16 040–33 719)	22 142 (16 040–46 171)	22 671 (18 473–32 688)	.5697^#^
Estrone (pmol/kg)	3744 (2847–9899)	7855 (3062–18 998)	3744 (2514–7415)	.1611
Estradiol (pmol/kg)	2320 (1537–4160)	2747 (1796–2937)	2306 (1537–4160)	.4211
Ratio cortisol:cortisone	4.13 (2.47–15.09)	42.28 (15.09–56.09)	3.35 (2.26–5.54)	<.0001
Ratio estradiol:estrone	0.46 (0.32–0.66)	0.54 (0.15–0.65)	0.44 (0.33–0.63)	.6059

*Student t-test *P*-values calculated with log-transformed variables. ^#^Satterthwaite adjusted *P*-value

*HSD11B1* mRNA abundance was positively correlated with the cortisol:cortisone ratio (*r* = 0.49; *P* = .0198) and negatively associated with relative cortisone amount (*r* = –0.49; *P* = .0221). In women with ER+/PR+ tumor, *HSD17B12* transcript amount (*r* = 0.58; *P* = .0479) was correlated with relative E1 amount in breast adipose tissue. *HSD17B12*, *HSD17B7*, or *CYP19A1* were not correlated with relative E2 amount. Women with cancer had higher expression of *HSD17B12* mRNA than controls (*P* = .0231). Relative E2 amount was positively associated with *ESR2* (ERβ) mRNA expression (*r* = 0.61; *P* = .0358), but not *ESR1* (ERα) mRNA expression in women with ER+/PR+ tumor. In women with breast cancer, abundance of *CYP19A1* transcript was positively correlated with *HSD11B1* mRNA expression (*r* = 0.54; *P* = .0326).

### Adiposity

**Figure 2.**
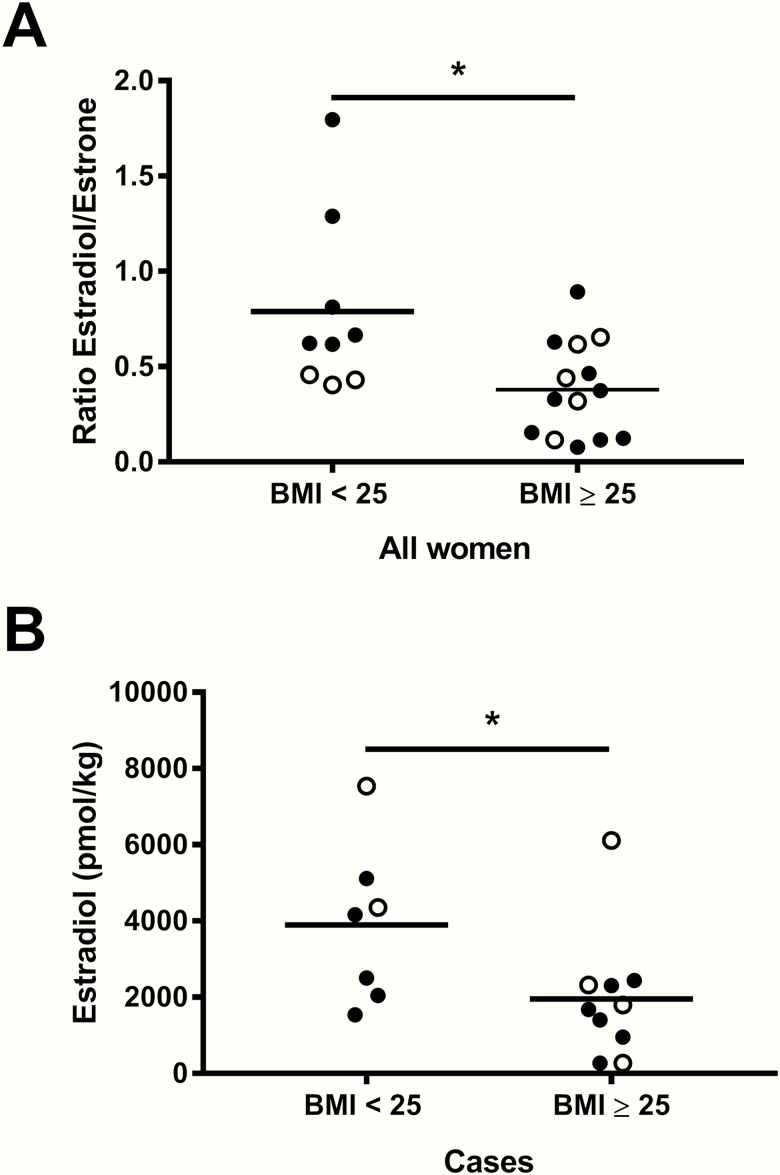
Adiposity and estrogens in breast adipose tissue. (A) Difference of the estradiol:estrone ratio according to body mass index (BMI) status using 25 kg/m^2^ as a cut-off (*P* = .0335, n = 23). (B) Difference of relative estradiol amounts in women with cancer according to BMI status using 25 kg/m^2^ as a cut-off (*P* = .0494, n = 17). Data on graphs are mean. Open circles represent premenopausal women data points and filled circles represent postmenopausal women data points. *P < .05.

The ratio of E2 to E1 was higher in lean women than in women with a BMI ≥ 25 kg/m^2^ (*P* = .0335) (Fig. 2A) (postmenopausal, n = 15, *P* = .0072; premenopausal, n = 8, *P* = NS) in the entire cohort, even if *CYP19A1* expression was higher in women with a BMI ≥ 25 kg/m^2^ (*P* < .05). This difference remained significant when considering only cancer cases (*P* = .0393, n = 17), or only ER+/PR+ patients (*P* = .0436, n = 13). The difference, although the same magnitude as above, was no longer statistically significant when considering ER+/PR+ patients with invasive carcinoma (stage ≥ 1) (*P* = .0900, n = 11). Relative E2 amount was higher in lean women than in women with obesity and overweight in cancer cases (*P* = .0494) (Fig. 2B) (postmenopausal, n = 11, *P* = .0325; premenopausal, n = 6, *P* = NS). cancer cases (*P* = .0494) (Fig. 2B). There was no difference in cortisol and cortisone between lean women and women with obesity and overweight (data not shown).

### Breast cancer clinical features

**Figure 3.**
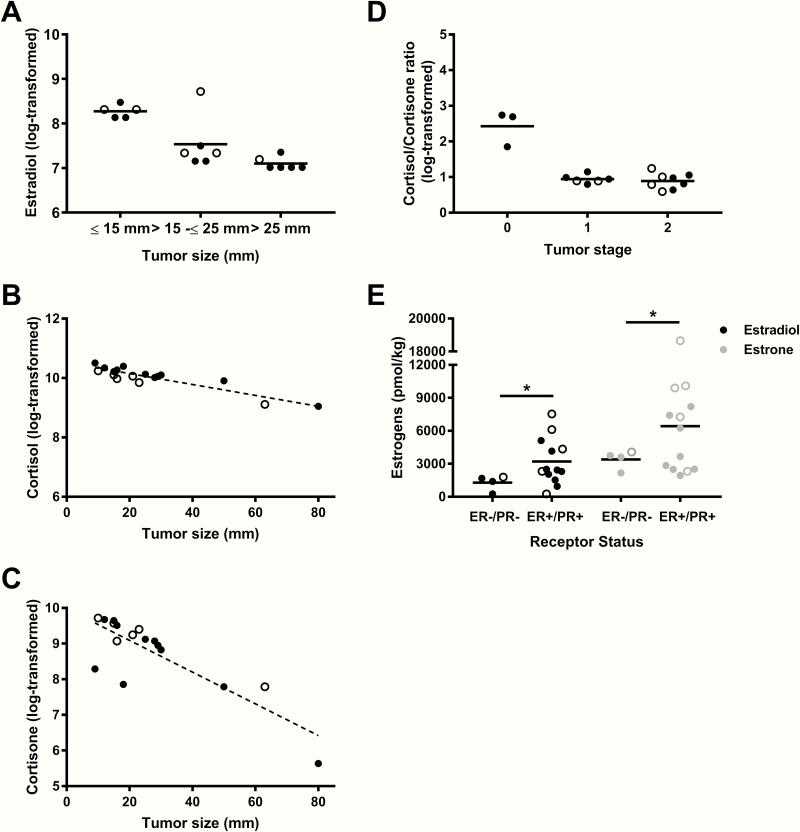
Breast cancer clinical features and relative breast adipose tissue steroid amounts. (A) Mixed-model regression between relative estradiol amount (log-transformed) and tertiles of tumor size adjusted for menopausal status and current intake of hormonal derivatives (β _2_ = –0.9785, β _3_ = –1.1197*; P* = .0488). (B) Mixed-model regression between relative cortisol amount (log-transformed) and tumor size (continuous) adjusted for body mass index (BMI), menopausal status, and current intake of hormonal derivatives (β = –0.02135, *P* = .0027). (C) Mixed-model regression between relative cortisone amount (log-transformed) and tumor size (continuous) adjusted for BMI, menopausal status, and current intake of hormonal derivatives (β = –0.03636, *P* < .0001). (D) Mixed-model between cortisol:cortisone ratio (log-transformed) and tumor stage adjusted for BMI (β _2_ = –1.8720, β _3_ = –1.8316; *P* = .0410). (E) Difference in relative estradiol and estrone amounts in ER–/PR– vs ER+/PR+ women (*P* = .0134 and *P* = .0454). Data on graphs are mean. n = 17. Open circles represent premenopausal women data points and filled circles represent postmenopausal women data points. *P < .05.

Relative adipose tissue E2 amount (log-transformed) was inversely associated with tumor size (categorical variable) (Fig. 3A) (*P* = .0281, n = 17) (postmenopausal, n = 11, *P* = .0283; premenopausal, no convergence), but this relationship did not reach statistical significance when tumor size was treated as a continuous variable (*β* = –0.0199, *P* = .1089, n = 17). Further adjustment for menopausal status and use of hormonal derivatives did not alter the results (*P* = .0488, n = 17) whereas the relationship was no longer significant after adjusting for BMI (*P* = .1569, n = 17) or when considering only patients with invasive carcinoma (stage ≥ 1) (*P* = .1157, n = 14). Including only women with ductal carcinoma generated similar effect size. Although the adjusted models were no longer significant, the nonadjusted model remained significant (data not shown). *CYP19A1* mRNA expression was positively correlated with tumor size (*r* = 0.49; *P* < .05), whereas expression of *ESR1* (ERα) mRNA was negatively associated with this variable (*r* = –0.54; *P* = .0296). There was no relationship between relative adipose tissue estrogen amounts and tumor stage or tumor grade in our sample.

The ratio of cortisol to cortisone was lower in women with cancer than in control women (*P* < .0001). This difference was driven by higher relative cortisone amount and not lower relative cortisol amount. In fact, relative cortisone amount was higher in cancer patients than in control patients (*P* = .0055). These differences remained when combining controls with women with ER–/PR– breast cancer who shared similar characteristics (Table 5), and comparing them with women with ER+/PR+ tumor (*P* = .0097 and *P* = .0171, respectively) (data not shown).

**Table 5.** Clinical characteristics of the women with ER–/PR– breast cancer and control women.

Variables (median, [Q1-Q3])	Control (n = 6)	ER–/PR– (n = 4)
Age (years)	53.7 (44.9–57.5)	54.9 (53.6–60.29)
BMI (kg/m^2^)	27.1 (24.3–29.4)	26.0 (25.5–26.6)
Premenopausal n (%)	2 (33)	1 (25)
Postmenopausal n (%)	4 (67)	3 (75)

Abbreviations: BMI, body mass index; ER, estrogen receptor; PR, progesterone receptor.

No difference was detected between control women and cancer-positive women with respect to cortisol (data not shown). Both log-transformed cortisol and cortisone amounts were inversely associated with tumor size (*β* = – 0.01873, *P* = .0007) and (*β* = –0.05048, *P* < .0001) independent of BMI, menopausal status, and current use of hormonal derivatives (*β* = –0.02135, *P* = .0027) and (*β* = –0.03636, *P* < .0001) (Fig. 3B and 3C) when tumor size was treated as a continuous variable. When we stratified according to menopausal status, the mixed-models remained significant for postmenopausal women (n = 11) (Fig. 3B and 3C; *P* = .0055, *P* < .0001) but did not converge for premenopausal women (n = 6). Those relationships were still significant when including only patients with invasive tumors (cortisol: *β* = –0.02376, *P* = .0078; *β*_*adj*_ = –0.02977, *P* = .0079, and cortisone: *β* = –0.03328, *P* < .0001; *β*_*adj*_ = –0.03650, *P* < .0001). Contrary to estradiol, cortisone and cortisol were not associated with tumor size (all TNM stages included or with only invasive tumors) (*P* = .0904 and *P* = .1894). Tumor stage was negatively associated with cortisol:cortisone ratio independent of BMI (β_2_ = –1.8720, β_3_ = –1.8316; *P* = .0410) (Fig. 3D) (postmenopausal, n = 11, *P* = .0660; premenopausal, n = 6, *P* = .3096). In sensitivity analyses, including only women presenting a ductal carcinoma histology phenotype, the models generated similar results (data not shown). There was no relationship between relative adipose tissue GC amounts and tumor grade in our sample.

Higher relative amounts of E2 and E1 were detected in adipose tissue of women with ER+/PR+ tumor than in women with ER–/PR– tumor (*P* = .0163 and *P* = .0134, respectively) (Fig. 3E) (postmenopausal, n = 11, *P* = .1077 and *P* = .4324, respectively; premenopausal, no convergence).

## Discussion

To our knowledge, this is the first study to report a LC-MS/MS quantification method combining analysis of cortisone, cortisol, E1, and E2 extracted from breast adipose tissue from both healthy women and women with breast cancer. Several lines of evidence showed that adipose tissue might play an active role in tumor initiation and progression (18, 36, 37). As such, the notion of an active cross-talk between adipose and tumor cells has been put forward in the literature (15, 38, 39). In vitro uptake of E1, E2, and cortisol from culture media by female abdominal adipose tissue explants was reported as being more than 2-fold lower than progesterone and testosterone, highlighting the possible contribution of steroid conversion in adipose tissue as a source of estrogens and GC for the tumor (40). Yet, the contribution of adipose tissue to relative steroid hormone amounts and their possible actions remain to be fully elucidated. Most of the steroidogenic pathways have been studied directly in breast tumors or in vitro. Reports of endogenous steroid hormones in breast adipose tissue mostly comprise analyses of estrogens (E1 and E2) and their corresponding fatty acyl-esters as well as androgen precursors, namely androstenedione and testosterone (41–44). Of note, a recent study characterized more than 20 steroids, including androgens, progestogens, and estrogens in breast adipose tissue by GC-MS/MS (45). A limitation of that study is the lack of data regarding normal breast adipose tissue from healthy controls.

### Quantification of steroids

We have been able to quantify these 4 steroids in most of our breast adipose samples. Interestingly, we found similar ranges in relative amounts for estrogens as those reported by Honma and collaborators in breast cancer tissue, using LC-MS/MS (46). These findings suggest that breast adipose tissue is a potent source of sex hormones for the tumor. One of the strengths of our study is the use of stable isotope dilution LC-MS/MS instead of the historically used ELISAs (enzyme-linked immunosorbent assays). ELISAs for steroid measurements have several drawbacks such as nonspecific antibody interactions, inconsistent reproducibility, and inadequate sensitivity (47). Moreover, they usually require separate assays for each compound of interest, demanding a large quantity of tissue. Using 3 stable isotope-labelled standards in our protocol allowed us to normalize for loss of analytes during the extraction process.

Contrary to our hypothesis, our data, reported as pmol/kg of whole adipose tissue, showed a decrease in the ratio of E2:E1 and a decrease of E2 with increasing adiposity, as assessed with the BMI, suggesting little impact of aromatase conversion per mass unit in adipose tissue. This can also be explained by the higher affinity of androstenedione as a substrate for aromatase compared with testosterone, as previously reported (48). Marchand et al. reported that higher circulating E2 concentration is directly associated with increased fat mass (49). Simpson and colleagues reported that increased aromatization in obesity was due to a higher number of cells and not to higher conversion activity per adipose tissue mass unit (50). Our results are also consistent with another study where the authors found a positive correlation between the E1:E2 ratio in visceral fat and BMI in postmenopausal women (51). Contrary to Savolainen-Peltonen, we found that *HSD17B12* mRNA expression is higher in adipose tissue from cancer patients than in controls (44). We cannot exclude that the lower relative amount of E2 observed in adipose tissue of overweight and obese women are due to increased uptake by the tumor cells as previously proposed by Savolainen-Peltonen et al. (44). However, we observed this difference in our entire sample and with all women with cancer, including those with ER– status. Effect size were not modified when stratification by ER+/PR+ was performed.

We were unable to find a significant association between BMI and relative cortisol amount in breast adipose tissue. We acknowledged that this may be due to our limited sample size. However, we found lower and nonquantifiable cortisone amount in breast adipose tissue of our control women (4 out of 6). Our group of control women had higher mean adipocyte size than our women with cancer. This is not surprising, as our control women tended to have higher BMI than the women with cancer, although not statistically significant probably because of our small sample. The adipocyte size difference reported between pre- and postmenopausal women is similar to the findings of Iyengar and collaborators (19). The lower amount of cortisone could be partially explained by a higher activation rate of cortisol or a lower inactivation of cortisol to cortisone by 11β-HSD1, because our gene expression results suggest that *HSD11B1* expression is positively associated with the cortisol:cortisone ratio and negatively associated with the cortisone relative amount. These results are consistent with previous findings from our team which showed that 11β-HSD1 activity and expression is positively associated with adipocyte size, at least in the abdominal subcutaneous and omental depots (24, 25). A previous study with obese subjects undergoing bariatric surgery has indeed found lower amount of cortisone in adipose tissue of obese subjects before weight loss and when compared with control with no difference in adipose tissue relative cortisol amount among those groups (52). No difference in tissue relative amount of cortisol could be explained by concomitant higher clearance by 5α-reductase, also increased in obesity (53). However, our data showed no decrease in cortisone with higher BMI, which suggests a different catabolism of cortisol and cortisone in women with breast cancer.

Some steroids were previously proposed to be increased in adipose tissue during obesity such as cortisol, which could also act as an immune suppressor in breast tissue. As reported by Cirillo et al., many different tumor types produce active cortisol which inhibits tumor-specific CD8+ T proliferation in vitro (54). Infiltration of CD8+ cells was linked to improved cancer-specific survival by Mohammed and collaborators (55). However, breast cancer was one of the types of cancer not showing any difference between *HSD11B1/2* expression between cancer and matched normal epithelial tissues, which suggest a paracrine, possibly by adjacent adipose tissue, instead of an autocrine effect by cortisol (54). In the same order of ideas, immunohistochemistry of 11β-HSD1 showed a presence of the enzyme in 64% of breast tumors and 97% of matched adjacent tissue (56) and GR protein amount was higher in breast tumor than in normal epithelial tissue (57). Increases in GC, particularly cortisol, can induce aromatase expression via the GRE on exon I.4 (26). On the other hand, cortisol via binding to GR is an activator of the estrogen sulfotransferase, which inactivates estrogens by adding a sulfate group and limiting its binding to ER (58).

We did observe higher relative cortisone amount in adipose tissue from women with cancer with no change in cortisol. As per our initial hypothesis, we did observe a decrease in relative estradiol, cortisol, and cortisone amounts according to tumor size and a decrease in cortisol:cortisone ratio with increasing tumor stage. As stated previously, the decrease in estradiol, but not estrone, with tumor size could represent an increased uptake by the tumor cells (43). The higher cortisol:cortisone ratio in patients with cancer than in control women and the further decrease of this ratio with tumor stage point to a dual effect of GCs in breast cancer related to stage of the disease. Increases in cortisol:cortisone ratio at the lower stages could increase estrogen production via activation of aromatase, but at a later stage, decreases in cortisol:cortisone could be explained by a negative feedback loop through increased estrogen production by the tumor and a concomitant lower expression of *HSD11B1*. However, we were unable to demonstrate a relationship between cortisol:cortisone ratio with E2:E1 ratio or E2 and E1 in our sample, contrary to previous results in visceral adipose tissue, but similar to results in subcutaneous tissue (51).

Despite our relatively low number of patients in both groups, we found that median E2 adipose tissue level was higher in women ER+/PR+ than ER–/PR–. Hennig et al. reported higher adipose tissue E2, androstenedione, and androsterone levels in women with ER+ breast cancer than those ER–, but no difference in E1 or any of the other androgens and progestogens (45). Falk et al. measured adipose tissue sex steroids by radioimmunoassay and found a significant difference only for testosterone and a trend for higher E2, E1, and androstenedione concentration in ER+/PR+ than in ER–/PR– samples (41). We found that relative E2 amount was associated with *ESR2* (ERβ) mRNA, but not that of *ESR1* (ERα) in women with ER+/PR+ tumor. *ESR2* expression is known to be increased with E2 production, not *ESR1*, either suggesting a negative feedback or no effect depending on depot origin (59). *ESR2* is only expressed in mature adipocytes in adipose tissue whereas *ESR1* is present in both the stromal vascular fraction and in mature adipocytes (60). Lower *ESR1* expression has been linked with adipose tissue dysfunction (61). However, a relationship with adipocyte cell size and *ESR1* expression was not observed in our study.

Limitations of our study are the relatively low number of participants which is counterbalanced by the wide range of BMI and prognostic marker values of our cohort. Of note, our total sample number is in the same order of magnitude as previous literature on breast adipose tissue steroid measurements (41–44), representing the difficulty in obtaining those samples for research purposes. Moreover, we have included in our analysis, control samples, namely samples from healthy women.

Data on steroid concentration in breast adipose tissue from healthy control and women with cancer are scarce in the literature. Most publications investigated this relationship in postmenopausal women (41, 42, 44) with the exception of Hennig et al. (45), who did include premenopausal women (n = 6 out of 51) in their analyses. They did not, however, separate according to menopausal status due their low number of premenopausal women. We acknowledge the potential difference in hormone metabolism between pre- and postmenopausal women. Because the main source of estrogens shifts from gonads to peripheral tissues in menopause, we adjusted for menopausal status and age in our analysis. We also performed stratification to further alleviate concerns about the influence of menopausal status, but these analyses should be interpreted with caution. It should be noted that inference in our study is limited to postmenopausal women, as our number of premenopausal women did not allow us to investigate fully their steroid concentration as a separate group. Most of our findings were still significant when including only postmenopausal women. Of note, tissue steroid concentration in our study did not vary as a function of menopause. Our cohort represents normal demographics in breast cancer, that is, increased prevalence in older, postmenopausal women. Hence, we suggest that these findings are relevant in this context.

The use of a cross-sectional design does not allow for causal inferences and we acknowledge that there might be reverse causality as higher tumor stage and size may cause changes in steroid metabolism rather than the opposite. Another constraint is that we cannot quantify the distance from the tumor at which the adipose tissue samples were taken. However, as the defined margins were all included in paraffin blocks for clinicopathological assessment directly at the hospital, we can attest that our adipose samples were taken at least 1 cm from the tumor extremities. Hennig et al. reported no difference between steroid concentration between 2 sample locations (<0.5 cm and >5 cm), except for E2 (45). Owing to the standard of care and acceptable limit margins in Quebec for clinicopathological assessment, it was not possible to include adipose tissue <0.5 cm away from the tumor in a research project. Therefore, the variation of E2 due to the relative distance from the tumor was likely limited.

## Conclusion

We were able to quantify estrogens and GCs in breast adipose tissue from both healthy women and women suffering from breast cancer. There is clear indication that steroid hormone metabolism is different among those 2 subgroups. Moreover, relative amounts of sex steroids in adipose tissue appear to be related to BMI, especially for E2, whereas differences in relative GC amounts appear to be more closely related to cancer progression. As such, relative estradiol amount was lower in women with larger tumors independently of age and menopausal status and relative GC amounts were negatively associated with tumor size, independently of age, menopausal status and BMI.

## Abbreviations

11β-HSD1/2: 11β-hydroxysteroid dehydrogenase type 1 and 2
^13^C_3_E1: 2,3,4-[^13^C_3_]-estrone
^13^C_3_E2: 2,3,4-[^13^C_3_]-17β-estradiol
17β-HSD: 17β-hydroxysteroid dehydrogenase
AR: analytical reagent
BMI: body mass index
CD8^+^ T: cytotoxic T cell
D_4_F: [^2^H_4_]-cortisol
DCIS: ductal carcinoma in situ
E1: estrone
E2: estradiol
ELISA: enzyme-linked immunosorbent assay
ER: estrogen receptor
ERβ: estrogen receptor beta
ESI: electrospray ionization
EtOAc: ethyl acetate
EtOH: ethanol
FA: formic acid
GC: glucocorticoid
GC-MS/MS: gas chromatography tandem mass spectrometry
GR: glucocorticoid receptor
GRE: glucocorticoid response element
HER2: human epidermal growth factor receptor 2
HPLC: high-performance liquid chromatography
HRT: hormonal replacement therapy
IDC: invasive ductal carcinoma
IS: internal standard
KS: Kolmogorov–Smirnov
LC-MS/MS: liquid chromatography tandem mass spectrometry
LLOQ: lower limit of quantitation
MeOH: methanol
MPPZ: 1-(2,4-dinitrophenyl)-4,4-dimethylpiperazinium
MRM: multiple reaction monitoring
OFN: oxygen-free nitrogen
PCR: polymerase chain reaction
PPZ: 1-(2,4-dinitro-5-fluorophenyl)-4-methylpiperazine
PR: progesterone receptor
UHPLC: ultrahigh-performance liquid chromatography
WC: waist circumference

## Additional Information

***Disclosure Summary:*** A.T. is the recipient of research grant support from Johnson & Johnson Medical Companies, Medtronic, and Pfizer for studies unrelated to this publication. The authors have nothing to disclose.

***Data Availability:*** Restrictions apply to the availability of data generated or analyzed during this study to preserve patient confidentiality or because they were used under license. The corresponding author will on request detail the restrictions and any conditions under which access to some data may be provided.
